# Minimal clinically important difference in Alzheimer's disease: Rapid review

**DOI:** 10.1002/alz.13770

**Published:** 2024-04-01

**Authors:** Ryan T. Muir, Michael D. Hill, Sandra E. Black, Eric E. Smith

**Affiliations:** ^1^ Department of Clinical Neurosciences and Department of Community Health Sciences Calgary Alberta Canada; ^2^ Hotchkiss Brain Institute University of Calgary Calgary Alberta Canada; ^3^ Division of Neurology Department of Medicine Sunnybrook Health Sciences Centre Toronto Ontario Canada; ^4^ L.C Campbell Cognitive Neurology Research Unit Dr Sandra Black Centre for Brain Resilience and Recovery, and Hurvitz Brain Sciences Program Sunnybrook Research Institute Toronto Ontario Canada

**Keywords:** Alzheimer's disease, clinical trial, minimal clinically important difference

## Abstract

**INTRODUCTION:**

We conducted a rapid systematic review of minimal clinically important differences (MCIDs) for Alzheimer's disease (AD) trial endpoints.

**METHODS:**

Two reviewers searched EMBASE, MEDLINE, and PubMed from inception to June 4, 2023.

**RESULTS:**

Ten articles were retrieved. For mild cognitive impairment (MCI), a change of +2 to +3 points on the Alzheimer's Disease Assessment Scale–Cognitive Subscale (ADAS‐Cog), +1 points on the Clinical Dementia Rating scale sum of boxes (CDR‐SB), −5 points on the integrated Alzheimer's Disease Rating Scale (iADRS), or −1 to −2 points on the Mini‐Mental State Examination (MMSE) was considered meaningful. For patients with mild AD, a change of +3 on the ADAS‐Cog, +2 points on CDR‐SB, −9 points on the iADRS, or −2 points on the MMSE was considered meaningful. For patients with moderate to severe AD, a change of +2 points on the CDR‐SB or a change of −1.4 to −3 points on the MMSE was considered meaningful.

**CONCLUSION:**

This review identified previously published MCIDs for AD trial endpoints. Input from patients and caregivers will be needed to derive more meaningful endpoints and thresholds.

**Highlights:**

- This systematic rapid review identified thresholds for minimal clinically important differences (MCIDs) for recently used Alzheimer's disease (AD) trial endpoints: Alzheimer's Disease Assessment Scale–Cognitive Subscale (ADAS‐Cog), Clinical Dementia Rating scale sum of boxes (CDR‐SB), integrated Alzheimer's Disease Rating Scale (iADRS), Mini‐Mental State Examination (MMSE).
- MCIDs were higher for more severe stages of AD.
- Average treatment effects in recent trials of anti‐amyloid disease modifying monoclonal antibodies are lower than previously published MCIDs.
- In future trials of disease modifying treatments for AD, the proportion of participants in each treatment group that experienced a clinically meaningful decline could be reported.
- More work is needed to incorporate the values and preferences of patients and care partners in deriving MCIDs.

## 1 BACKGROUND

The minimal clinically important difference (MCID) has been defined as the smallest unit of change in an outcome measure that would be perceived as a difference that is clinically meaningful for patients, caregivers, or health practitioners, and would constitute the grounds for change in a patient's care.^1^ Knowing the MCID can help trialists design studies powered to detect meaningful change and help clinicians and patients interpret the results of trials.

It has been proposed that the MCID can be defined through both *anchor‐based* and *distribution‐based* methods. Anchor‐based MCID methodology examines the association between scores on an administered instrument and another independent measure, such as clinician ascertainment of meaningful change, which is used as the anchor of clinical meaningfulness.^1^ The two requirements of an anchor‐based approach are that the anchor must be interpretable and, second, that there must be an association between the target and the anchor. Distribution‐based methods express the clinical effect in terms of the underlying distribution of the measure.^1^ However, a consensus group recommends that a distribution‐based method, which considers only measurement properties and not relationship to patient‐reported outcomes, should not be used to derive an MCID.^2^ Regulatory authorities such as the U.S. Food and Drug Administration also prefer anchor‐based methods to define MCIDs.^3^

The clinical meaning of Alzheimer's disease (AD) trial endpoints has become a topic of intense discussion after two trials demonstrated that anti‐beta‐amyloid monoclonal antibodies reduce the rate of cognitive and functional decline in patients with mild cognitive impairment (MCI) and mild dementia due to AD.^4^, ^5^ Some editorialists have questioned whether the reduced rates of decline compared to placebo were clinically meaningful^6^, ^7^ and justified the cost. In this context, we undertook this rapid review to systematically identify previous studies designed to derive or validate MCIDs for AD trials.

## 2 METHODS

A systematic review was conducted, following the Cochrane Rapid Review Methods Group methods for Rapid Reviews.^8^ We first published the review protocol on the Vascular Cognitive Impairment Research Group of the University of Calgary on June 29, 2023 (https://cumming.ucalgary.ca/labs/smith‐research/research‐studies) and subsequently registered it on Open Science Framework (doi:10.17605/OSF.IO/DQ3VC) on August 15, 2023.

To be included, studies were required to have a main study objective of comparing or deriving MCIDs for a pre‐specified set of AD trial endpoints, using anchor‐based or distribution‐based methods. AD of any stage was allowed, except preclinical AD^9^ (ie, normal cognition with positive AD biomarkers was not included). Biomarker support for the diagnosis of AD was not required. MCIDs for the following endpoints, used in recent phase 3 trials in AD, were pre‐specified as being of interest: Folstein Mini‐Mental State Examination (MMSE),^10^ Clinical Dementia Rating scale sum of boxes (CDR‐SB),^11^ Alzheimer's Disease Cooperative Study Activities of Daily Living (ADCS‐ADL) scale,^12^ ADCS‐MCI‐ADL scale,^13^ ADCS Instrumental ADL scale (ADCS‐iADL),^12^ Alzheimer's Disease Assessment Scale–Cognitive Subscale (ADAS‐Cog)^14^ and variants, and the integrated Alzheimer's Disease Rating Scale (iADRS).^15^ Conference proceedings, abstracts, posters, letters to the editor or opinion pieces, studies not published in English, and studies including patients without AD were excluded.

**RESEARCH IN CONTEXT**

1. **Systematic review**: Two reviewers searched EMBASE, MEDLINE, and PubMed from inception to June 4, 2023, using search terms related to “Alzheimer Disease” and “minimal clinically important difference.” We did not find previously published systematic reviews on this subject.
2. **Interpretation**: This systematic review returned 10 relevant articles that provided minimal clinically important differences (MCIDs) for mild cognitive impairment (MCI), mild, moderate, and severe Alzheimer's disease (AD) with dementia for the Alzheimer's Disease Assessment Scale–Cognitive Subscale (ADAS‐Cog), Clinical Dementia Rating scale sum of boxes (CDR‐SB), integrated Alzheimer's Disease Rating Scale (iADRS), and Mini‐Mental State Examination (MMSE). MCIDs for more severe stages of AD tended to be higher. Previously published MCIDs were larger than the treatment effect observed in recent trials of anti‐amyloid disease‐modifying monoclonal antibodies for AD.
3. **Future directions**: More work is needed to incorporate the values and preferences of patients and care partners in deriving MCIDs. Although average treatment effects in recent trials were lower than the MCIDs, minorities of patients may have experienced clinically meaningful benefits. In future trials of disease‐modifying treatments for AD, the proportion of participants in each treatment group that experienced a clinically meaningful decline should be reported.

EMBASE, MEDLINE and PubMed were searched from database inception to June 4, 2023. The search terms were developed from identified key articles and in consultation with each database's respective subject headings. Search terms for key words in all text fields and subject headings included (“Alzheimer Disease”) AND (“minimal clinically important difference” OR “clinically meaningful difference” OR “meaningful change” OR “minimum detectable change” OR “minimum detectable difference” OR “clinically meaningful” OR “clinically important” OR “meaningful decline”). The full search strategies are displayed in Supplementary Tables S1‐S3. Prior to initiating screening for study eligibility, a pilot review of titles and abstracts by RTM and EES suggested that the search had sufficient sensitivity as articles previously known to be relevant were captured.

Two reviewers (RTM and EES) independently screened titles and abstracts and subsequently selected articles for full text review using web‐based software (Covidence, Melbourne, Australia). Conflicts at title and abstract screening and full text review were resolved by consensus. To identify other potentially relevant articles, the references of articles selected for full text review were also screened by both reviewers and relevant references also screened for inclusion in the full text review as well.

Data were extracted from full text articles using an electronic data collection form (Covidence, Melbourne, Australia). Data extraction occurred in duplicate with a primary reviewer (RTM) extracting data and a second reviewer (EES) evaluating for correctness and completeness. Extracted information included year of publication, sample size, mean age, percent female, stage of AD as defined by the study, study design, whether an anchor‐ or distribution‐based method was used, and whether the study employed triangulation of anchor‐ and distribution‐based methods to arrive at a single recommended MCID. In this review, we use the terms “mild AD,” “moderate AD,” and “severe AD” to refer to stages of dementia due to AD.

Study quality was assessed using a subset of questions extracted from the COnsensus‐based Standards for the selection of health Measurement INstruments (COSMIN) risk of bias tool.^16^ Studies were considered at risk for bias if any questions were scored as “inadequate” or “doubtful”; however, studies were not excluded based on quality assessment. The quality of the source data for MCID derivation was assessed using the Newcastle‐Ottawa scale for cohort and case‐control studies,^17^ Cochrane Risk of Bias Assessment 2 for Randomized Control Trials,^18^ or the Joanna Briggs Institute Critical Appraisal Checklist for systematic reviews,^19^ as appropriate. A single reviewer rated risk of bias initially (RTM) with verification and validation by a second reviewer (EES).

Because of the small numbers of studies returned for each MCID, we did not attempt to do meta‐analyses or analyses for publication bias.

## 3 RESULTS

**FIGURE 1:**
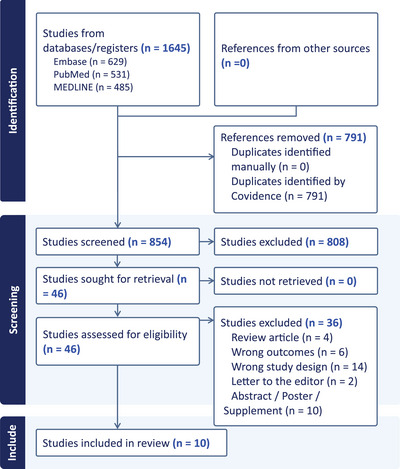
Study selection.

The results of the literature search are shown in Figure 1. After removing duplicates, the search of PubMed, Medline, and EMBASE yielded 854 articles which were screened by abstract and 46 articles which were read in full by two reviewers, resulting in 10 articles which by consensus met the criteria for eligibility.

Characteristics of the 10 included studies are shown in Table 1. Two studies validated a previously derived MCID for ADAS‐Cog by comparing it to anchors, while the other eight studies derived their own MCID thresholds. There were four articles that provided anchor‐based MCIDs for MMSE; four that provided anchor‐based MCIDs for ADAS‐Cog; two that provided anchor‐based MCIDs for CDR‐SB; one that provided an anchor‐based MCID for iADRS; and one that provided only measures of reliability and error of measurement for CDR‐SB and MMSE, which were framed as distribution‐based MCIDs. Triangulated MCIDs incorporating multiple sources of information were provided for MMSE (four studies), CDR‐SB (two studies), and iADRS (one study). Data sources included randomized controlled trials (RCTs), a non‐randomized clinical trial, a systematic review of RCTs, cohort studies (Alzheimer's Disease Neuroimaging Initiative [ADNI] and the National Alzheimer Coordinating Center [NACC] Uniform Data Set [UDS]), an expert survey, an expert consensus meeting, and a systematic review. Industry fully funded three of the studies, partially funded two others, and did not fund five.

**TABLE 1:** Design of the included studies.

Author and year	MCID metric	Method	Data source	Industry funded	Longitudinal	Risk of bias
Burback 1999^20^	MMSE	Anchor	Expert survey	No	N/A	Low
Rockwood 2007^21^	ADAS‐Cog	Anchor	Single arm trial (ACADIE^22^)	Partial	Yes (6 months)	Low
Rockwood 2010^23^	ADAS‐Cog	Anchor	RCT (VISTA^24^)	Partial	Yes (6 months)	Low
Howard 2011^25^	MMSE	Anchor Distribution Triangulation	RCT (DOMINO^26^)	No	Yes (12 months)	High
Schrag 2012^27^	ADAS‐Cog	Anchor Distribution Triangulation	ADNI^28^	No	Yes (6 months)	Low
Andrews 2019^29^	CDR‐SB MMSE	Anchor Distribution Triangulation	NACC UDS^30^, ^31^	Yes	Yes	High
Watt 2021^32^	ADAS‐Cog MMSE	Distribution	Systematic review RCTs	No	Yes (variable)	Low
Borland 2022^33^	MMSE	Anchor Distribution Triangulation	Cohort (BIOFINDER^34^)	No	Yes (mean 19 months)	Low
Wessels 2022^35^	iADRS	Anchor Distribution Triangulation	RCT (AMARANTH^36^ and EXPEDITION3^37^)	Yes	Yes (12 or 18 months)	Low
Lansdall 2023^38^	ADAS‐Cog CDR‐SB MMSE	Anchor Distribution Triangulation	RCT (ADC‐008^39^)	Yes	Yes (12 months)	Low

Abbreviations: ADAS‐Cog, Alzheimer's Disease Assessment Scale–Cognitive Subscale^14^; ADNI, Alzheimer's Disease Neuroimaging Initiative; iADRS, integrated Alzheimer's Disease Rating Scale; CDR‐SB, Clinical Dementia Rating scale sum of boxes^11^; MCID, minimal clinically important difference; MMSE, Mini‐Mental State Examination; NACC UDS, National Alzheimer Coordinating Center Uniform Data Set; RCT, randomized controlled trial.^10^

Study quality was generally good (Tables S4‐S7). However, one study^29^ was deemed to be at risk for bias because the meaning of the anchor, a clinician impression of meaningful decline, was not clear, and another study^25^ was deemed at risk for bias because it was based on expert consensus but without a recorded method and without specification of the number of experts or their disciplines.

The characteristics of the study populations in which the MCIDs were derived are shown in Table 2. For the studies that derived MCIDs using RCT data or cohort studies, the mean ages ranged from 70.7 to 76.7, and 42.1% to 72% were women. AD stages varied by study (see Table 2) and included MCI, mild dementia, moderate dementia, and severe dementia.

**TABLE 2:** Population characteristics of the included studies.

Author and year	AD stage	N	Age	Percent women
Burback 1999^20^	Mild to severe AD	161	NR	NR
Rockwood 2007^21^	Mild to mod AD	95	75.8 (7.7)	72%
Rockwood 2010^23^	Mild to mod AD (MMSE 10‐25)	99	76.7 (7.3)	58%
Howard 2011^25^	Mod‐severe AD (MMSE 5‐15)	127	NR	NR
Schrag 2012^27^	Mild AD (MMSE 20‐26)	181	75.2 (7.5)	47%
Andrews 2019^29^	MCI Mild AD (MMSE 20‐26) Mod‐severe AD (MMSE < 20)	19,566^a^	73.1 (9.8)	56.9%
Watt 2021^32^	Mild to severe AD	NR	NR	NR
Borland 2022^33^	MCI	292	71.1 (5.5)	42.1%
Wessels 2022^35^	MCI Mild AD (MMSE 20‐26)	4347	70.7 to 72.7 (SD 6.6 to 7.8)^b^	51.5% to 57.9%^b^
Lansdall 2023^38^	MCI	769	72.9 (7.3)	46%

Abbreviations: AD, Alzheimer's disease with dementia; MCI, mild cognitive impairment; MMSE, Mini‐Mental State Examination^10^; mod, moderate; NR, not reported.

^a^ For overall population, including cognitively unimpaired subjects not analyzed in this systematic review.

^b^ Pooled analysis of three trials; for age and percent women, the range across trials is shown.

Table 3 shows MCID thresholds by anchor‐based methods. The studies determined MCIDs using standard methods.^40^ First, it was verified that the outcome scale was correlated with the chosen anchor. Next, patients were grouped based on a clinically relevant threshold on the chosen anchor (for example, one point or greater change in the Global Deterioration Scale). Finally, statistical analyses such as linear regression were used to estimate the difference in the outcome measure that corresponded to the anchor change. Anchors varied considerably across studies, consisting of measures of functional change, hybrid cognitive and functional scores (CDR global score or CDR‐SB), clinician or caregiver and clinician global impressions of change, clinician‐judged worsening, or expert opinion. Most studies used a clinician‐based anchor of meaningful change, and only two^27^, ^38^ considered patient or caregiver‐based anchors. MCID thresholds were lowest for MCI and were higher for more advanced stages of disease. Many studies reported MCID thresholds rounded to the nearest whole unit change on the scale, suitable for assigning meaningful change for each individual patient, while others provided MCIDs as averages with decimal places.

**TABLE 3:** MCID thresholds derived using anchor or anchor‐informed triangulation.

Stage	Author	MCID threshold	Method	Anchor
**ADAS‐Cog**				
MCI	Lansdall^38^	+4.95 (6.73)	Anchor	GDS −1 pt at 36 months
		+9.01 (8.41)	Anchor	GDS −2 pt at 36 months
		+1.41 (4.15)	Anchor	MCI‐CGIC “minimal” at 12 months
		+3.60 (5.09)	Anchor	MCI‐CGIC “moderate” at 12 months
		+2	Triangulation	Minimal deterioration
		+3 to +4	Triangulation	Moderate deterioration
Mild AD	Schrag^27^	+3.8 (2.1, 5.5)	Anchor	Clinician‐judged worsening on memory testing
		+3.6 (1.9, 5.3)	Anchor	Clinician‐judged worsening on non‐memory testing
		+3.5 (1.9, 5.0)	Anchor	Clinician‐judged worsening on FAQ
		+3.1 (1.5, 4.8)	Anchor	Clinician‐judged worsening on total CDR rating
		+3.98 (4.82)	Anchor	Change in global CDR ≥1 stage
		+3	Triangulation	
**ADAS‐Cog13**				
MCI	Lansdall^38^	+6.04 (8.16)	Anchor	GDS −1 pt at 36 months
		+10.58 (9.24)	Anchor	GDS −2 pt at 36 months
		+1.84 (4.71)	Anchor	MCI‐CGIC “minimal” at 12 months
		+4.36 (5.71)	Anchor	MCI‐CGIC “moderate” at 12 months
		+2	Triangulation	Minimal deterioration at 12 months
		+4	Triangulation	Moderate deterioration at 12 months
**CDR‐SB**				
MCI	Andrews^29^	+0.98	Anchor	Clinician‐recorded meaningful decline
		+1	Triangulation	
	Lansdall^38^	+1.08 (1.18)	Anchor	GDS −1 pt at 12 months
		+3.39 (1.92)	Anchor	GDS −2 pt at 12 months
		+0.64 (1.02)	Anchor	MCI‐CGIC “minimal” at 12 months
		+2.35 (1.66)	Anchor	MCI‐CGIC “moderate” at 12 months
		+1	Triangulation	Minimal deterioration at 12 months
		+2.5	Triangulation	Moderate deterioration at 12 months
Mild AD	Andrews^29^	+1.63	Anchor	Clinician‐recorded meaningful decline
		+2	Triangulation	
Mod‐Sev AD	Andrews^29^	+2.3	Anchor	Clinician‐recorded meaningful decline
		+2	Triangulation	
**iADRS**				
MCI	Wessels^35^	−5.5 (−7.1, −3.9)	Anchor	CDR‐SB +1 pt/year
	(AMARANTH)	−3.5 (−5.4, −1.5)	Anchor	MMSE −1 pt/year
		−5.2 (−7.2, −3.2)	Anchor	FAQ +3 pt/year
		−5	Triangulation	
Mild AD	Wessels^35^ (AMARANTH)	−9.5 (−11.2, −7.8)	Anchor	CDR‐SB +2 pt/year
		−5.9 (−7.5, −4.3)	Anchor	MMSE −2 pt/year
		−7.5 (−9.2, −5.7)	Anchor	FAQ +3 pt/year
	Wessels^35^ (EXPEDITION3)	‐12.2 (−14.2, −10.2)	Anchor	CDR‐SB +2 pt/18 months
		−7.0 (−8.3, −5.7)	Anchor	MMSE −2 pt/year
		−7.2 (−8.8, −5.7)	Anchor	FAQ +3 pt/year
	Wessels^35^	−9	Triangulation	
**MMSE**				
MCI	Andrews^29^	−1.26 (−1.33, −1.20)	Anchor	Clinician‐recorded meaningful decline
		−2.55 (−2.76, −2.34)	Anchor	CDR global change ≥1 stage
		−1	Triangulation	
	Borland^33^	−1.9 (−2.2 to −1.5)	Anchor	CDR‐SB change ≥0.5
		−2.4 (−2.9 to −2.0)	Anchor	CDR‐SB change ≥1
		−1.7	Triangulation	
	Lansdall^38^	−2.62 (3.41)	Anchor	GDS −1 pt at 36 months
		‐6.56 (4.81)	Anchor	GDS −2 pt at 36 months
		−2 to −3	Triangulation	Minimal deterioration
		−6 to −7	Triangulation	Moderate deterioration
Mild AD	Andrews^29^	−2.32 (−2.41, −2.24)	Anchor	Clinician‐recorded meaningful decline
		−2.35 (−2.55, −2.16)	Anchor	CDR global change ≥1 stage
		−2	Triangulation	
Mild‐Sev AD	Burback^20^	−3.72 (−3.95, −3.50)	Anchor	Clinician survey
Mod‐Sev AD	Andrews^29^	−3.22 (−3.37, −3.06)	Anchor	Clinician‐recorded meaningful decline
		−4.99 (−5.77, −4.22)	Anchor	CDR global change ≥1 stage
		−3	Triangulation	
	Howard^25^	−1 to −2	Anchor	Expert consensus
		−1.4	Triangulation	

*Note*: Values are mean (SD) or mean (5% confidence limit, 95% confidence limit).

Abbreviations: AD, Alzheimer's disease with dementia; ADAS‐Cog, Alzheimer's Disease Assessment Scale‐Cognitive 11 item^14^; ADAS‐Cog13, ADAS‐Cog 13 item derivative^41^; CDR, Clinical Dementia Rating scale^11^; CDR‐SB, Clinical Dementia Rating scale sum of boxes^11^; FAQ, Functional Activities Questionnaire^42^; GDS, Global Deterioration Scale^43^; iADRS, integrated Alzheimer's Disease Rating scale^15^; MCI, mild cognitive impairment; MCI‐CGIC, Mild Cognitive Impairment Clinical Global Impression of Change^44^; MCID, minimal clinically important difference; MMSE, Mini‐Mental State Examination^10^; Mod, moderate; pt, point; Sev, severe.

For the ADAS‐Cog, there were studies in MCI and mild AD (Table 3). A study in patients with MCI provided MCIDs of +2 for minimal deterioration and +3 to +4 for moderate deterioration,^38^ while a study in mild AD provided an MCID of +3.^27^ For the 13‐item derivative of the ADAS‐Cog, MCIDs of +2 for minimal deterioration and +4 to +5 for moderate deterioration were provided.^38^ Two more studies did not derive their own threshold for ADAS‐Cog (Table 4), but instead looked at the validity of an ADAS‐Cog threshold of +4 which has been previously proposed for RCTs^21^, ^23^ (Table 4). In both studies, the ADAS‐Cog threshold of +4 was only mildly to moderately correlated with anchors. The ADAS‐Cog 4‐point threshold had 22% to 55% sensitivity and 81% to 89% specificity for worsening based on patient and caregiver attainment goals and global impression of change.

**TABLE 4:** Studies validating a previously derived MCID threshold.

Stage	Author & year	MCID	Anchor	Associations with MCID threshold
Mild to Mod AD	Rockwood 2007^21^	ADAS‐Cog +4	PGAS	Spearman *r *= −0.15
			CGAS	Spearman *r *= −0.27
			CIBIC‐Plus	Spearman *r *= +0.38
			MMSE	Spearman *r *= −0.24
			PGAS worse	Sens 22%, Spec 76%, PPV 9%, NPV 90%
			CGAS worse	Sens 41%, Spec 81%, PPV 39%, NPV 82%
			CIBIC‐plus worse	Sens 38%, Spec 83%, PPV 52%, NPV 72%
	Rockwood 2010^23^	ADAS‐Cog +4	PGAS worse	Sens 55%, Spec 89%, PPV 37%, NPV 94%
			CGAS worse	Sens 43%, Spec 88%, PPV 38%, NPV 90%
			CIBIC‐plus worse	Sens 24%, Spec 88%, PPV 50%, NPV 70%

Abbreviations: AD, Alzheimer's disease with dementia; ADAS‐Cog, Alzheimer's Disease Assessment Scale‐Cognitive^14^; CGAS, Clinician Goal Attainment Scale^22^; CIBIC‐Plus, Clinician's Interview‐Based Impression of Change‐Plus Caregiver Input^45^; MCID, minimal clinically important difference; MOD, moderate; NPV, negative predictive value; PGAS, Patient/caregiver Goal Attainment Scale^22^; PPV, positive predictive value; Sens, sensitivity; Spec, specificity.

For the CDR‐SB, there were studies in MCI, mild AD, and moderate to severe AD (Table 3). For MCI, one study provided an MCID of +1,^29^ while another study provided MCIDs of +1 for minimal deterioration and +2.5 for moderate deterioration.^38^ For mild AD, the MCID was +2.^29^ For moderate to severe AD, the MCID was +2.^29^

For the iADRS, one study provided MCID estimates for MCI (−5) and mild AD (−9) (Table 3).^35^

For the MMSE, for MCI three studies provided MCIDs of −1,^29^ −1.7,^33^ and −2 to −3.^38^ For mild AD the MCID was −2,^29^ and for AD dementia of any stage the MCID was −3.72.^20^ For moderate to severe AD the MCID was −3^29^ and −1.4,^25^ based on two studies.

The results of measures of distribution, responsiveness, and reliability to change are provided in Table 5. Some studies provided measures in the whole study population, while others provided them only in subgroups that met an anchor threshold. Most studies reported 0.5 or 0.4 SD of the baseline distribution as one of the distribution measures, but a variety of other metrics were also reported.

**TABLE 5:** Distribution and responsiveness measures.

Stage	Author	Anchor	Distribution method	Value
**ADAS‐Cog**				
MCI	Lansdall^38^		0.5 SD of baseline	2.19
			SEM of baseline	2.57
			S_diff_	3.63
			RCI	7.12
Mild AD	Schrag^27^	Clin‐judged worsening on memory testing	ES	0.6
			0.5 SD of baseline	3.4
			SRM of baseline	0.7
		Clin‐judged worsening on non‐memory testing	ES	0.5
			0.5 SD of baseline	3.6
			SRM of baseline	0.6
		Clin‐judged worsening on FAQ	ES	0.5
			0.5 SD of baseline	3.3
			SRM of baseline	0.7
		Clin‐judged worsening on total CDR	ES	0.4
			0.5 SD of baseline	3.6
			SRM of baseline	0.6
		CDR global change ≥1 stage	ES	0.70
			SRM of baseline	0.83
Mild‐Sev AD	Watt^32^		0.5 SD of baseline	5.0
			0.4 SD of baseline	4.0
			0.5 SD of change	3.2
			0.4 SD of change	2.6
**ADAS‐Cog13**				
MCI	Lansdall^38^		0.5 SD of baseline	3.04
			SEM of baseline	2.98
			S_diff_	4.21
			RCI	8.25
**CDR‐SB**				
MCI	Andrews^29^		ES	0.44
			SRM	0.57
			0.5 SD of baseline	1.12
	Lansdall^38^		0.5 SD of baseline	0.39
			SEM of baseline	0.45
			S_diff_	0.63
			RCI	1.24
Mild AD	Andrews^29^		ES	0.54
			SRM	0.70
			0.5 SD of baseline	1.52
Mod‐Sev AD	Andrews^29^		ES	0.51
			SRM	0.81
			0.5 SD of baseline	2.25
**iADRS**				
MCI	Wessels^35^	CDR‐SB +1 pt/year	ES	−0.57
	(AMARANTH)		SRM	−0.70
			0.5 SD of baseline	4.84
		MMSE −1 pt/year	ES	−0.42
			SRM	−0.42
			0.5 SD of baseline	4.17
		FAQ +3 pt/year	ES	−0.60
			SRM	−0.66
			0.5 SD of baseline	4.34
Mild AD	Wessels^35^	CDR‐SB +2 pt/year	ES	−0.83
	(AMARANTH)		SRM	−1.09
			0.5 SD of baseline	5.76
		MMSE −2 pt/year	ES	−0.58
			SRM	−0.62
			0.5 SD of baseline	5.10
		FAQ +3 pt/year	ES	−0.59
			SRM	−0.93
			0.5 SD of baseline	6.32
Mild AD	Wessels^35^	CDR‐SB +2 pt/18 months	ES	−1.07
	(EXPEDITION3)		SRM	−1.06
			0.5 SD of baseline	5.74
		MMSE −2 pt/year	ES	−0.56
			SRM	−0.72
			0.5 SD of baseline	6.21
		FAQ +3 pt/year	ES	−0.54
			SRM	−0.74
			0.5 SD of baseline	6.70
**MMSE**				
MCI	Borland^33^		ES	0.8
			SEM	−1.4
			RCI	3
	Andrews^29^		ES	−0.86
			SRM	−0.80
			0.5 SD of baseline	1.48
	Landsall^38^		0.5 SD of baseline	0.92
			SEM of baseline	1.33
			S_diff_	1.89
			RCI	3.70
Mild AD	Andrews^29^		ES	−0.80
			SRM	−0.69
			0.5 SD of baseline	1.48
Mild‐Sev AD	Watt^32^		0.5 SD of baseline	2.0
			0.4 SD of baseline	1.6
			0.5 SD of change	1.8
			0.4 SD of change	1.4
Mod‐Sev AD	Howard^25^		0.5 SD of baseline	2.1
			0.4 SD of baseline	1.7
			0.5 SD of change	1.7
			0.4 SD of change	1.4
Mod‐Sev AD	Andrews^29^		ES	−1.12
			SRM	−1.08
			0.5 SD of baseline	2.23

*Note*: If not otherwise specified, values are for the whole study population. Some studies reported distribution and responsiveness measures in subgroups meeting specific anchor thresholds. Abbreviations: AD, Alzheimer's disease with dementia; ADAS‐Cog, Alzheimer's Disease Assessment Scale‐Cognitive 11 item^14^; ADAS‐Cog13, ADAS‐Cog 13 item derivative^41^; CDR, Clinical Dementia Rating scale; CDR‐SB, Clinical Dementia Rating Scale Sum of Boxes; Clin., clinician; ES, effect size (mean difference/baseline SD); FAQ, Functional Activities Questionnaire^42^; MCI, mild cognitive impairment; Mod, moderate; MMSE, Mini‐Mental State Examination^10^; RCI, reliable change index calculated as 1.96*SEM√2; SD, standard deviation; SEM, standard error of the mean calculated as SD*√(1‐Pearson correlation); S_diff_, standard error of difference calculated as SEM√2; Sev, severe; SRM, standardized response mean (mean difference/SD of change).

## 4 DISCUSSION

**FIGURE 2 alz13770-fig-0002:**
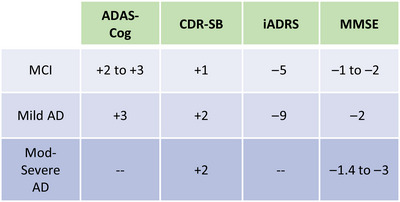
Summary of minimal clinically important differences for Alzheimer's disease trial endpoints. Values indicate the minimal clinically important difference (MCID) for decline. AD, Alzheimer's disease; ADAS‐Cog, Alzheimer's Disease Assessment Scale‐Cognitive^14^; CDR‐SB, Clinical Dementia Rating scale sum of boxes^11^; iADRS, integrated Alzheimer's Disease Rating scale^15^; MCI, mild cognitive impairment; MMSE, Mini‐Mental State Examination^10^; Mod, moderate.

The following MCID thresholds have some support in the literature (Figure 2). For MCI, a change of +2 to +3 points on ADAS‐Cog, +1 point on CDR‐SB, −5 points on iADRS, or −1 to −2 points on MMSE could be considered meaningful decline. For patients with mild AD, a change of +3 on ADAS‐Cog, +2 points on CDR‐SB, −9 points on iADRS, or −2 points on MMSE could be considered meaningful decline. For patients with moderate to severe AD, a change of +2 points on CDR‐SB or a change of −1.4 to −3 points on MMSE could be considered meaningful decline; there were no published studies providing MCIDs for ADAS‐Cog or iADRS for moderate to severe AD.

Anchors for MCIDs varied considerably. They included global assessment of function, clinician impression of change with or without caregiver input, and clinician impression informed by functional scales. One study derived their MCID from an expert survey and another from the consensus of a panel of clinical trialists. None included direct input from persons with lived experience in the determination of MCIDs.^16^ This raises the question of which is the most robust method to derive an anchor and whether an anchor should be derived from both clinician and patient or caregiver ascertainments of meaningful change. Furthermore, there is inherent circularity in the derivation of some of the MCIDs for AD outcome metrics using other AD outcome metrics as anchors.

Consensus group and regulatory authorities recommend the use of anchor‐based over distribution‐based methods for determining MCIDs.^3,^ ^40^ The main criticism of the distribution‐based approach is that it does not consider clinician, patient, or caregiver ascertainments of meaningfulness. Distribution‐based approaches are best viewed as a hedge that a change in score is not due to chance or measurement error.

The MCIDs were derived from studies of varying duration (6 to 18 months; Table 1), which may have introduced heterogeneity. Additionally, this is relatively short follow‐up for a long‐term disease process, and additional studies using anchors derived from longer term follow‐up would be useful. Because less effective treatments will need to be applied for a longer duration to achieve a meaningful change, more research is needed on how the required duration of treatment affects patient and caregiver perception of meaningfulness.

The concept of clinically important differences in AD has been much discussed after RCTs showed that two beta‐amyloid immunotherapies are highly effective at removing brain amyloid but only partly effective at reducing cognitive and functional decline.^4^, ^5^ Editorialists have noted that the mean difference in rate of decline between treatment and placebo is less than previously published MCID thresholds.^6^, ^7^ In the CLARITY‐AD trial of lecanemab, the mean decline among placebo‐treated participants at 18 months was +1.66 points on the CDR‐SB, exceeding the MCID for MCI (+1) but not for mild AD with dementia (+2), and the difference with treatment was −0.45 points.^4^ Thus, many placebo‐treated patients did not experience a meaningful decline over 18 months and the average group difference was less than the MCID. In the TRAILBLAZER‐ALZ2 trial of donanemab, the mean decline among placebo‐treated participants at 18 months on the iADRS was −13.11, which exceeds the MCID for MCI (−5) and mild AD (−9), but the difference with treatment was only 2.92.^5^ Therefore, on average there was a clinically meaningful decline, but the average group difference was not clinically meaningful. By comparison, trials of cholinesterase inhibitors showed differences of −2.57 points on the ADAS‐Cog,^46^ a mean difference that is also less than the MCID for ADAS‐Cog (+3).[Fig alz13770-fig-0002]

It may, however, be premature to conclude that the effects of these beta‐amyloid immunotherapies are not clinically meaningful. The use of average differences obscures the possibility that a significant minority of treated patients may have had a clinically meaningful response. To better understand the results, the trials should report the proportion of patients in the drug and placebo groups that had a clinically meaningful decline, by applying MCID thresholds to each individual patient. For example, in TRAILBLAZER‐ALZ2 cumulative incidence curves showed that approximately 37% of placebo‐treated patients had an increase in CDR global stage of ≥0.5 at 18 months compared with 25% of donanemab‐treated patients, for an absolute risk difference of 12%.^5^ CDR global stages, which were used as an anchor for several of the studies identified by this review (Table 3), correspond roughly to questionable, mild, moderate, and severe dementia.^11^ Reporting the proportion of patients who exceed an MCID change threshold over a given time period allows for calculation of risk ratios and numbers needed to treat, which are staple metrics of evidence‐based medicine and would be more familiar to clinicians than mean differences in scales used predominantly for research. For donanemab, approximately 8.3 patients would need to be treated for 18 months to prevent one patient from having a meaningful decline in CDR global stage (hazard ratio 0.63, 95% confidence interval, 0.51 to 0.77).^5^ This approach is supported by analogy to the stroke literature where extracting the clinical meaning based upon average post‐stroke functional disability scores is similarly difficult and the field has adopted the reporting of the proportion of patients who achieve a certain functional disability threshold, most commonly using the modified Rankin Score.^47^

Another method for expressing the clinical meaning of AD treatments has been the “time saved approach.”^48^ In this approach, estimates of least‐square means from regression models are used to determine the extra time it would take the treated group to decline to the level of the placebo group. For donanemab, the time saved was 5.3 months; that is, at the end of 18 months of treatment a donanemab‐treated patient experienced only the amount of decline of a placebo‐treated patient at 12.7 months.^48^ However, we did not find any studies that derived an MCID for time saved.

MCIDs will vary across patients and clinicians depending on their values. Goal attainment scaling, in which the participants themselves identify their own individual anchors for treatment effects, has been proposed as a method to capture individually meaningful treatment effects in Alzheimer's disease.^49^ Most studies on MCIDs were published prior to 2020, well before disease modifying therapies became a reality, and reflect clinician values in an earlier, less hopeful era. If, at that time, clinicians expected that only large changes were meaningful then MCIDs may be overestimated. Based on their primary endpoints, lecanemab reduced the rate of decline in AD by 27.1% and donanemab by 22.3%. One might ask whether, in retrospect, it was unrealistic to expect larger relative effects on decline. In many domains of medicine, a 20% to 30% relative reduction is considered a realistically achievable and important therapeutic effect. It has been pointed out that the average effects in the CLARITY‐AD and TRIALBLAZER‐ALZ2 studies may cross accepted MCID thresholds if the treatment effects are extended to 36 months or longer.^50^ However, this relies on extrapolating linear effects for much longer than the trials’ actual duration.

The MCID does not address the value of a new treatment in terms of cost effectiveness. A clinically meaningful treatment may nonetheless not be desirable from a health systems standpoint because of cost. Conversely, a treatment that fails to exceed a threshold for clinical meaning for clinicians and patients may nonetheless be valuable to a health system if it reduces costs with acceptable risks. For these reasons, it will be critical to analyze and model healthcare costs of persons treated with AD disease modifying therapies.

This rapid review is, of course, limited by the existing literature. We found only 10 relevant studies. Most studies (8 of 10) were judged to be high quality with low risk for bias. There is a need for more studies that are contemporary and that include input from persons with lived experience. Because perceptions of cognitive disability are strongly influenced by culture, race/ethnicity, and gender, it will be important to seek diverse input on the meaningfulness of trial endpoints. MCIDs in AD outcomes were derived with respect to meaningful difference in rate of decline; whether these differences are also relevant to therapies that stabilize or improve cognitive function is unknown and should be explored in future studies.

In conclusion, this systematic review identified previously published MCIDs for clinical trial endpoints used recently in AD disease modifying trials of beta‐amyloid targeting monoclonal antibodies. These were based on a relatively small number of studies without direct patient and caregiver input. In our view, the controversy over the clinical relevance of recently published disease modifying therapies for AD should prompt trialists and regulatory authorities to consider dichotomizing AD trial endpoints based on clinically meaningful change thresholds, reporting risk differences and number needed to treat. These metrics may be more understandable and meaningful to patients and clinicians than mean differences on research scales that are little used in routine practice, or office‐based cognitive tests, that do not directly measure daily function. More research is needed on determining clinically meaningful change, directly incorporating the values of persons with lived experience.

## CONFLICT OF INTEREST STATEMENT

Dr. Muir and Dr. Hill report no relevant conflicts of interest. Dr. Black reports supervising contracted research (fees paid to her institution) funded by GE Healthcare, Genentech, Optina, Roche, Eli Lilly, Eisa/Biogen Idec, Novo Nordisk, Lilly Avid; personal consulting fees from Roche, Biogen, Novo Nordisk, Eisai, and Eli Lilly; and payments or honoraria from Biogen, Roche, and Eisai. Dr. Smith has done personal consulting for Alnylam Pharmaceuticals and Eli Lilly and has served on an advisory board for Eisai (unpaid). Author disclosures are available in the supporting information.

## CONSENT STATEMENT

Informed consent was not required because this was a systematic review, without interaction with human subjects.

## Supporting information

Supporting Information

Supporting Information
