# Supplementary material for: Minimal clinically important difference in Alzheimer's disease: Rapid review
Source: Alzheimers Dement. 2024 Apr 1;20(5):3352–63. doi: 10.1002/alz.13770 (PMC11095473; doi:10.1002/alz.13770)
Supplement: Supplementary file 1 — Supporting Information [file ALZ-20-3352-s002.docx]

**Supplemental files**

**Supplemental Table S1: MEDLINE search strategy**

|  | **TERMS** | **Number of Records** |
| --- | --- | --- |
| 1 | exp Alzheimer Disease/ | 117566 |
| 2 | Alzheimer's.mp. or exp Cognitive Dysfunction/ | 190057 |
| 3 | minimal clinically important difference.mp. or exp Minimal Clinically Important Difference/ | 3308 |
| 4 | minimal important difference.mp. | 565 |
| 5 | minimal clinically relevant change.mp. | 7 |
| 6 | minimum detectable change.mp. | 561 |
| 7 | minimum detectable difference.mp. | 99 |
| 8 | meaningful change.mp. | 1508 |
| 9 | clinically meaningful difference.mp. | 351 |
| 10 | clinical outcome assessment.mp. | 267 |
| 11 | clinically important.mp. | 28755 |
| 12 | clinically meaningful.mp. | 14866 |
| 13 | meaningful decline.mp. | 89 |
| 14 | 1 or 2 | 214479 |
| 15 | 3 or 4 or 5 or 6 or 7 or 8 or 9 or 10 or 11 or 12 or 13 | 45109 |
| 16 | 14 and 15 | 485 |

**Supplemental Table S2: EMBASE search strategy**

|  | **TERMS** | **Number of Records** |
| --- | --- | --- |
| 1 | Alzheimer disease.mp. or exp Alzheimer disease/ | 254500 |
| 2 | exp minimal clinically important difference/ | 4445 |
| 3 | minimal important difference.mp. | 1013 |
| 4 | minimal clinically relevant change.mp. | 9 |
| 5 | exp minimum detectable change/ | 1113 |
| 6 | minimum detectable difference.mp. | 139 |
| 7 | meaningful change.mp. | 2675 |
| 8 | clinically meaningful difference.mp. | 718 |
| 9 | clinical outcome assessment.mp. | 467 |
| 10 | clinically important.mp. | 42042 |
| 11 | clinically meaningful.mp. | 28811 |
| 12 | meaningful decline.mp. | 163 |
| 13 | 2 or 3 or 4 or 5 or 6 or 7 or 8 or 9 or 10 or 11 or 12 | 73152 |
| 14 | 1 and 13 | 629 |

**Supplementary Table S3: Pubmed Search Strategy**

| **Search Terms** | **Number of Records** |
| --- | --- |
| ("Alzheimer disease"[MeSH Terms] OR "Cognitive Dysfunction"[MeSH Terms] OR ("Alzheimer"[All Fields] AND "disease"[All Fields]) OR "Alzheimer disease"[All Fields] OR Alzheimer's[All Fields]) AND (minimal clinically important difference[MeSH] OR "minimal clinically important difference"[All Fields] OR "minimal important difference"[All Fields] OR "minimal clinically relevant change"[All Fields] OR "minimum detectable change"[All Fields] OR "minimum detectable difference"[All Fields] OR "meaningful change"[All Fields] OR "clinically meaningful difference"[All Fields] OR "clinical outcome assessment"[All Fields] OR "clinically important"[All Fields] OR "clinically meaningful"[All Fields] OR "meaningful decline"[All Fields]) | 531 |

**Supplemental Table S4. Study Quality According to COSMIN Checklist**

|  | Target  Population | Clear What Anchor Measures? | Anchor Measurement Properties | Anchor Statistical Method | Other Flaws? | Survey Method | All Disciplines? | Appropriate Number | Appropriate Analysis | At Least 2 Researchers? |
| --- | --- | --- | --- | --- | --- | --- | --- | --- | --- | --- |
|  | Box 1  Item 5 | Box 9  Item 1 | Box 9  Item 2 | Box 9  Item 3 | Box 9  Item 4 | Box 2  Item 22 | Box 2  Item 23 | Box 2  Item 24 | Box 2  Item 25 | Box 2  Item 26 |
| Burback 1999[1] | N/A | N/A | N/A | N/A | N/A | Very good | Adequate | Very good | Very good | Adequate |
| Rockwood 2007[2] | Adequate | Very good | Adequate | Very good | Very good | N/A | N/A | N/A | N/A | N/A |
| Rockwood 2010[3] | Very good | Very good | Adequate | Very good | Very good | N/A | N/A | N/A | N/A | N/A |
| Howard 2011[4] | N/A | N/A | N/A | N/A | N/A | Doubtful | Doubtful | Doubtful | Doubtful | Adequate |
| Schrag 2012[5] | Very good | Adequate | Very good | Very good | Very good | N/A | N/A | N/A | N/A | N/A |
| Andrews 2019[6] | Very good | Inadequate | Doubtful | Very good | Very good | N/A | N/A | N/A | N/A | N/A |
| Watt 2021[7] | N/A | N/A | N/A | N/A | Very good | N/A | N/A | N/A | N/A | N/A |
| Borland 2022[8] | Very good | Very good | Very good | Very good | Very good | N/A | N/A | N/A | N/A | N/A |
| Wessels 2022[9] | Very good | Very good | Very good | Very good | Very good | N/A | N/A | N/A | N/A | N/A |
| Lansdall 2023[10] | Very good | Very good | Very good | Very good | Very good | N/A | N/A | N/A | N/A | N/A |

**Table S4 Legend:** Study quality was assigned using relevant items from the the COnsensus-based Standards for the selection of health Measurement INstruments (COSMIN) risk of bias tool[11]. The first five criteria are relevant to studies where participant data were used to calculate the minimal clinically important difference (MCID), while the last five are relevant to studies where expert opinion was used to calculate the MCID. Most studies used appropriate methods and were at low risk of bias. One study^[6]^ used clinician impression of meaningful decline as the anchor, without further specification, which was deemed by the reviewers to have inadequate clarify regarding its meaning. One study[4] was based on expert consensus but without a recorded method and without specification of the number of participants or their disciplines.

**Supplemental Table S5. Quality of RCTs For Anchor-Based Measures**

|  | RCT | Sequence Generation | Allocation Concealment | Blinding  Participants/Personnel | Blinding Outcome | Incomplete Data | Selective Reporting | Other |
| --- | --- | --- | --- | --- | --- | --- | --- | --- |
| Rockwood 2010[3] | VISTA | Low | Low | Low | Low | Low | Low | Low |
| Howard 2011[4] | DOMINO[12] | Low | Low | Low | Low | High | Low | Low |
| Wessels 2022[9] | AMARANTH[13] | Low | Low | Low | Low | Low | Low | Low |
| Wessels 2022[9] | EXPEDITION-3[14] | Low | Low | Low | Low | Low | Low | Low |
| Lansdall 2023[10] | ADC-008[15] | Low | Low | Low | Low | Low | Low | Low |

**Table S5 Legend:** Four studies used data from five previously published randomized controlled trials to derive or validate minimal clinically important differences. To assess quality of these studies, we used the Cochrane Risk of Bias Assessment 2 for Randomized Control Trials[16]. “Low” indicates low risk of bias, and “High” indicates high risk of bias. One study was judged to have high risk of bias for incomplete data, because even the analysis was done according to the intent-to-treat principle the reported high rate of non-adherence was very high (39% did not adhere to study-assigned treatment).

**Supplemental Table S6. Quality of Cohort Studies For Anchor-Based Measures**

|  | Cohort | Exposed: Representative | Unexposed: Selection | Exposure: Ascertainment | Outcome Not Present At Start | Comparability | Outcome | Follow Up: Long Enough | Follow up: Completeness |
| --- | --- | --- | --- | --- | --- | --- | --- | --- | --- |
| Rockwood 2007[2] | ACADIE[17] | Truly | N/A | Structured Interview | Yes | N/A | Unsure | Yes | Bias unlikely |
| Schrag 2012[5] | ADNI[18] | Somewhat | N/A | Structured Interview | Yes | N/A | Unsure | Yes | Unsure |
| Andrews 2019[6] | NACC UDS[19, 20] | Somewhat | N/A | Structured Interview | Yes | N/A | Unsure | Yes | Unsure |
| Borland 2022[8] | BIOFINDER[21] | Somewhat | N/A | Structured Interview | Yes | N/A | Unsure | Yes | Unsure |

**Table S6 Legend:** Three studies used data from previously published longitudinal cohort studies and one study used data from a single arm unblinded trial**[2]** to derive or validate minimal clinically important differences (MCIDs). To assess quality of these studies, we used the Newcastle-Ottawa scale for cohort and case-control studies[22]. The exposed cohort was considered truly representative if it closely matched the population characteristics of clinical trial patients. Because for the MCID design there are no selected controls, we entered “N/A” for the scale item for selection of unexposed controls and for comparability of cases and controls. For the scale item on quality of the outcome assessment, we entered “Unsure” because none of the studies reported whether the MCID measure was assessed without knowledge of the anchor measure, and vice versa. N/A, not applicable.

**Supplemental Table S7. Quality of Systematic Reviews of Distribution-based Measures**

|  | Question Explicit | Inclusion Criteria | Search Strategy | Search Sources | Appraisal Criteria | Two Reviewers | Data Extraction | Pooling Methods | Publication Bias | Recommendations: Public/Practice | Recommendations: Research |
| --- | --- | --- | --- | --- | --- | --- | --- | --- | --- | --- | --- |
| Watt 2021[7] | Yes | Yes | Yes | Yes | Yes | Yes | Yes | Yes | Yes | N/A | Yes |

**Table S7 Legend:** Quality was assessed using a checklist published by the Joanna Briggs Institute[23]. For this systematic review, we considered recommendations for public policy and practice to be not applicable, because the main purpose of deriving minimal clinically important differences is to aid future research.

**Supplement - References**

[1] Burback D, Molnar FJ, St John P, Man-Son-Hing M. Key methodological features of randomized controlled trials of Alzheimer's disease therapy. Minimal clinically important difference, sample size and trial duration. Dementia and geriatric cognitive disorders. 1999;10:534-40.

[2] Rockwood K, Fay S, Gorman M, Carver D, Graham JE. The clinical meaningfulness of ADAS-Cog changes in Alzheimer's disease patients treated with donepezil in an open-label trial. BMC neurology. 2007;7:26.

[3] Rockwood K, Fay S, Gorman M. The ADAS-cog and clinically meaningful change in the VISTA clinical trial of galantamine for Alzheimer's disease. International journal of geriatric psychiatry. 2010;25:191-201.

[4] Howard R, Phillips P, Johnson T, O'Brien J, Sheehan B, Lindesay J, et al. Determining the minimum clinically important differences for outcomes in the DOMINO trial. International journal of geriatric psychiatry. 2011;26:812-7.

[5] Schrag A, Schott JM, Alzheimer's Disease Neuroimaging I. What is the clinically relevant change on the ADAS-Cog? J Neurol Neurosurg Psychiatry. 2012;83:171-3.

[6] Andrews JS, Desai U, Kirson NY, Zichlin ML, Ball DE, Matthews BR. Disease severity and minimal clinically important differences in clinical outcome assessments for Alzheimer's disease clinical trials. Alzheimers & Dementia 2019;5:354-63.

[7] Watt JA, Veroniki AA, Tricco AC, Straus SE. Using a distribution-based approach and systematic review methods to derive minimum clinically important differences. BMC Med Res Methodol. 2021;21:41.

[8] Borland E, Edgar C, Stomrud E, Cullen N, Hansson O, Palmqvist S. Clinically relevant changes for cognitive outcomes in preclinical and prodromal cognitive stages: Implications for clinical Alzheimer trials. Neurology. 2022;99:e1142-e53.

[9] Wessels AM, Rentz DM, Case M, Lauzon S, Sims JR. Integrated Alzheimer's Disease Rating Scale: Clinically meaningful change estimates. Alzheimer's & dementia (New York, N Y). 2022;8:e12312.

[10] Lansdall CJ, McDougall F, Butler LM, Delmar P, Pross N, Qin S, et al. Establishing clinically meaningful change on outcome assessments frequently used in trials of mild cognitive impairment due to Alzheimer's disease. The journal of prevention of Alzheimer's disease. 2023;10:9-18.

[11] Mokkink LB, de Vet HCW, Prinsen CAC, Patrick DL, Alonso J, Bouter LM, et al. COSMIN Risk of Bias checklist for systematic reviews of Patient-Reported Outcome Measures. Qual Life Res. 2018;27:1171-9.

[12] Jones R, Sheehan B, Phillips P, Juszczak E, Adams J, Baldwin A, et al. DOMINO-AD protocol: donepezil and memantine in moderate to severe Alzheimer's disease - a multicentre RCT. Trials. 2009;10:57.

[13] Wessels AM, Tariot PN, Zimmer JA, Selzler KJ, Bragg SM, Andersen SW, et al. Efficacy and Safety of Lanabecestat for Treatment of Early and Mild Alzheimer Disease: The AMARANTH and DAYBREAK-ALZ Randomized Clinical Trials. JAMA neurology. 2020;77:199-209.

[14] Honig LS, Vellas B, Woodward M, Boada M, Bullock R, Borrie M, et al. Trial of Solanezumab for Mild Dementia Due to Alzheimer's Disease. N Engl J Med. 2018;378:321-30.

[15] Petersen RC, Thomas RG, Grundman M, Bennett D, Doody R, Ferris S, et al. Vitamin E and donepezil for the treatment of mild cognitive impairment. N Engl J Med. 2005;352:2379-88.

[16] Sterne JAC, Savovic J, Page MJ, Elbers RG, Blencowe NS, Boutron I, et al. RoB 2: a revised tool for assessing risk of bias in randomised trials. BMJ. 2019;366:l4898.

[17] Rockwood K, Graham JE, Fay S, Investigators A. Goal setting and attainment in Alzheimer's disease patients treated with donepezil. J Neurol Neurosurg Psychiatry. 2002;73:500-7.

[18] Petersen RC, Aisen PS, Beckett LA, Donohue MC, Gamst AC, Harvey DJ, et al. Alzheimer's Disease Neuroimaging Initiative (ADNI): clinical characterization. Neurology. 2010;74:201-9.

[19] Morris JC, Weintraub S, Chui HC, Cummings J, Decarli C, Ferris S, et al. The Uniform Data Set (UDS): clinical and cognitive variables and descriptive data from Alzheimer Disease Centers. Alzheimer disease and associated disorders. 2006;20:210-6.

[20] Besser L, Kukull W, Knopman DS, Chui H, Galasko D, Weintraub S, et al. Version 3 of the National Alzheimer's Coordinating Center's Uniform Data Set. Alzheimer disease and associated disorders. 2018.

[21] Petrazzuoli F, Vestberg S, Midlov P, Thulesius H, Stomrud E, Palmqvist S. Brief Cognitive Tests Used in Primary Care Cannot Accurately Differentiate Mild Cognitive Impairment from Subjective Cognitive Decline. Journal of Alzheimer's disease : JAD. 2020;75:1191-201.

[22] Stang A. Critical evaluation of the Newcastle-Ottawa scale for the assessment of the quality of nonrandomized studies in meta-analyses. European journal of epidemiology. 2010;25:603-5.

[23] Aromataris E, Fernandez R, Godfrey CM, Holly C, Khalil H, Tungpunkom P. Summarizing systematic reviews: methodological development, conduct and reporting of an umbrella review approach. JBI Evidence Implementation. 2015;13:132-40.
